# Prevalence, Perception, and Predictors of Advance Directives among Hong Kong Chinese: A Population-Based Survey

**DOI:** 10.3390/ijerph16030365

**Published:** 2019-01-28

**Authors:** Carmen W. H. Chan, Martin M. H. Wong, Kai Chow Choi, Helen Y. L. Chan, Amy Y. M. Chow, Raymond S. K. Lo, Michael M. K. Sham

**Affiliations:** 1 The Nethersole School of Nursing, The Chinese University of Hong Kong, Hong Kong; martinwong@cuhk.edu.hk (M.M.H.W.); kchoi@cuhk.edu.hk (K.C.C.); helencyl@cuhk.edu.hk (H.Y.L.C.); 2 Department of Social Work and Social Administration, University of Hong Kong, Hong Kong; chowamy@hku.hk; 3 Geriatrics and Palliative Medicine, Shatin Hospital, Hong Kong; losk@ha.org.hk; 4 Bradbury Hospice, Hong Kong; 5 Palliative Medical Unit, Grantham Hospital, Hong Kong; shammk@ha.org.hk

**Keywords:** advance directives, advance care planning, end-of-life, population-based survey, telephone survey, Chinese

## Abstract

Advance directives (AD) can be used for the communication of healthcare decisions that may be required in the future when individuals have lost their capacity to make such decisions. The aim of this study is to examine the prevalence, perception, and predictors of AD completion in the Hong Kong general population with a diverse culture. Through random-digit dialing, a population-based telephone survey was conducted with participants aged 18 or above. Socio-demographic characteristics, self-perception and health status, prevalence of AD, and perceptions related to AD were assessed. The acceptance on completing AD was measured by the summed score on the level of agreement in making AD. In total, 2002 participants completed the survey, with only 0.5% having made AD. However, the majority of those who had heard about AD had made or intended to make AD (80.2%). Multivariable regression analysis showed that being religious, being optimistic, and agreeing to respect patients’ wishes are independently associated with higher AD acceptance. Being a student is associated with lower AD acceptance. The extremely low completion rate of AD, but high acceptance of AD urges for more active promotion of AD to the public and education on end-of-life care among university students.

## 1. Introduction

Patients are sometimes kept alive when they would prefer to die in peace, dignity, and comfort. For example, the application of mechanical ventilation during the advanced stage of a critical illness may extend a patient’s lifespan, but it may also prolong their suffering. Healthcare professionals often face difficult dilemmas in determining treatment choices for the very ill patients or those lacking the capacity to communicate treatment choices and without any previous evidence of their wishes. Personal preferences of end-of-life (EOL) decisions can be communicated in the form of written statements, known as advance directives (AD), concerning any healthcare decisions that may be required in the future when individuals have lost the capacity to make such decisions. The AD concept is largely derived from the principle of informed consent and a belief in personal autonomy regarding healthcare decisions. 

The completion rate of AD is generally low worldwide and it varies among different countries and regions. Despite the fact that AD legislation was passed in 1991, the completion rate of AD in the United States (US) is still low, with 26.3% of the general population having completed AD [1]. In the Netherlands, where two types of AD exist to refuse treatments or request euthanasia, only 7% of the Dutch population have completed AD [2]. The lack of awareness on AD was the most frequently reported reason for not having AD in both studies [1,2]. On the other hand, those who preferred AD thought that AD helps reduce end-of-life suffering [3]. Several studies pointed out that the difference of AD completion is related to ethnicity, with Caucasians being more likely to have completed AD than African Americans in the US [1,3,4,5,6,7,8]. Cultural differences were found to contribute a significant part to the variation in EOL decision making among regions [9,10]. 

There are several factors affecting the acceptance or completion of AD in the general population. Being female [3,11,12], of an older age [1,3,6,11,13], having higher education levels [1,2,3,14], and higher income [1,15] are associated with higher rates of acceptance or completion of AD in the community. For factors, such as religion, the findings seem to be culturally specific [16]. For example, being Jewish and Catholic was shown to predict AD completion in the US [3], but not having a religion was associated with higher AD completion rate in the Netherlands [2,11]. Regarding health status, those with poorer health status, such as having undergone major surgery and chronic use of medication, have higher rates of AD completion [3,14,17]. However, individuals perceiving themselves as having a poor health status are less eager to complete AD [18]. Overall, demographics, culture, and health status could have an effect on AD completion among populations.

As an international city with more than seven million people where “East meets West”, Hong Kong provides an ideal setting for investigating cultural-specific issues in a developed society, which serves as a model for other developed regions in the world. Although it is a former British colony, more than 90% of its population are Chinese [19], who emphasize family values and consider longevity a blessing and death as a big taboo [20]. With such cultural values, the EOL services for the public is a neglected area and its quality is poorer than expected. According to the 2015 Quality of Life Index published by the Economist Intelligence Unit, Hong Kong was ranked 22nd out of 80 regions among the globe, whereas the UK was ranked the highest [21]. In 2006, the discussion of AD in Hong Kong was initiated by the Government’s Law Reform Commission, and it was concluded that Hong Kong was not ready for a statutory form of AD [22]. Recently, Hospital Authority Palliative Care Services in Hong Kong advocated that patients suffering from life-limiting diseases should have the opportunity to participate in their care planning [23]. The AD form in Hospital Authority requires the signatures from two witnesses, in which one of them must be a physician, after thorough discussions. The common law in Hong Kong now recognizes AD as a legally bound document [24]. However, a recent telephone survey in Hong Kong revealed that only 14.3% of the population had heard of AD, but 73.9% of them agreed with the concept of AD after an explanation [25]. In contrast, a cross-national study revealed more Hong Kong students (49.4%) than US students (24%) preferred to extend their lives despite the pain and suffering [26]. Those with previous knowledge of do-not-attempt-cardiopulmonary-resuscitation expressed a higher preference to make AD [25]. To date, the prevalence and perception related to AD in the Chinese general population remains unknown, and further exploration of these issues is necessary to provide clues to the development of strategies in the promotion of the use of AD. The aim of this study is to examine the prevalence and perception of AD in the Hong Kong general population, identify its predictors, and clarify the public view of AD in a developed society with a diverse culture.

## 2. Materials and Methods

### 2.1. Study Design and Population

This is a cross-sectional, population-based telephone survey conducted in Hong Kong from September 2017 to March 2018. Participants who (1) were aged 18 or above, and (2) could communicate in Chinese were recruited and interviewed anonymously by trained interviewers from the Telephone Survey Research Laboratory, Hong Kong Institute of Asia-Pacific Studies, the Chinese University of Hong Kong. Random-digit dialing on fixed network numbers was employed as a strategy to enhance generalizability to the population and ensure that only Hong Kong residents were approached, as it is very common to have a fixed line for every household in Hong Kong. The interviews were conducted between 6:15 pm and 10:15 pm to avoid over-representation of the non-working population in the sample. In the case of households with more than one eligible participant, the participant whose birthday was closest to the interview date was invited to join the study. Participants would no longer be contacted if at least two unsuccessful attempts of calling were made at different time periods to ensure that the survey results were not biased by high non-response rates. Verbal informed consent was obtained from the participants after providing them with a description of the study. Ethical approval was obtained from the Joint Chinese University of Hong Kong-New Territories East Cluster Clinical Research Ethics Committee (Reference number: CRE-2013.133). 

### 2.2. Measurement

The questions in the survey were formulated by the research team based on the conceptual model developed by Chan et al [27]. To develop the survey, we conducted qualitative individual interviews with 96 participants divided into four groups, including patients with life-limiting diseases, their family members, healthcare professionals, and hospital volunteers, and a literature search to generate statements about the perception and influential factors related to AD [27]. The statements were then rated by the participants during the interviews and consolidated by the research team comprising two researchers in palliative care nursing and one researcher in social work specialized in palliative care, two palliative care physicians, and one statistician to ensure high face validity and content validity. The survey questionnaire is attached in Table S1. It consists of five parts: (1) Socio-demographic characteristics, (2) self-perception and health status, (3) prevalence of AD, (4) perceptions related to AD, and (5) level of agreement in making AD in various scenarios. It took 5 to 10 min to complete the survey.

Socio-demographic characteristics, including sex, age, education, marital status, employment status, religion, and monthly household income, were collected. Items pertaining to self-perception and health status, including self-rated health, ever had a serious disease or cancer, family history of serious diseases or cancers, playing a key role in family, self-perceived level of optimism, and level of agreement that patients’ wishes and decisions should be respected, were included in the survey. Prevalence of AD was examined by items enquiring the participants whether they had ‘ever heard about AD’, ‘had made AD’, and ‘intend to make AD’. A seven-point rating scale (1 = strongly disagree, 7 = strongly agree) was adopted in the section with items assessing perceptions related to AD and level of agreement in making AD in various scenarios, with ratings between five and seven being considered as agreeing or strongly agreeing with the statement indicated in each item. Items assessing perceptions related to AD included the level of agreement to the following statements: (1) AD is a basic human right, (2) AD should encompass basic nursing care, including pain relief and wound cleansing, (3) healthcare professionals should carry out AD by legislation, (4) adequacy of promotion on AD in the community, (5) patients should have a clear mind and be mentally prepared when considering making AD, (6) healthcare professionals should possess good communication skills when discussing AD completion with patients, (7) in addition to healthcare professionals and patients, family members of patient should be engaged in discussing AD completion, and (8) records throughout the processes of discussion and decision making on AD completion with patients should be kept. The acceptance on completing AD was measured by the summed score of the seven items in the section assessing the level of agreement in making AD in various scenarios, including: (1) When my health condition is too serious to be treated effectively, (2) in order to reduce the physical and psychological burden on my family members, (3) when considering the side effects of survival treatments and their adverse effects on quality of life, (4) if healthcare professionals can provide you with clear explanation and recommendation on AD, (5) if there is effective communication and coordination among healthcare professionals at different institutions to execute your decisions, (6) if you can have thorough discussions and follow-up with health professionals about AD, and (7) if AD is a legally bound instrument. Although the scale has fewer items (*n* = 7) compared with the Advance Directive Attitude Survey (ADAS) (*n* = 16) [28], it covers three out of four areas (impact of AD on the family, effect of an AD on treatment, and illness perception) of ADAS.

### 2.3. Statistical Analysis

The survey data are presented using appropriate descriptive statistics, in the form of a frequency (percentage) for categorical variables and a mean (standard deviation) for continuous variables. The internal consistency of the seven items assessing the acceptance on AD completion was examined by using Cronbach’s alpha. With the Cronbach’s alpha being 0.81, the survey exhibited a good internal consistency in assessing acceptance on AD completion. A summed score (between 0 and 100) was computed to quantify the degree of acceptance on AD completion, with a higher score indicating a higher degree of acceptance. In computing the summed score, responses including ‘don’t know’ or ‘unsure’ for the seven items assessing acceptance on AD completion (each assessed using a seven-point rating scale, 1 to 7) were regarded as missing values and were resolved by data imputation using the mean values of the remaining valid responses provided by at least four out of the seven statements having valid responses; otherwise the summed score was considered as missing and the subject concerned was not included in the subsequent association analysis. The raw summation score (RS) of the seven items therefore ranged from 7 to 49. The scores were then rescaled to the range of 0 to 100 by transformation using the formula: (RS − 7)/ (49 − 7). Univariate analyses and multivariable regression were then performed to identify factors significantly and independently associated with the summed score. All statistical tests involved were two-sided with the level of significance set at 0.05. Statistical analyses were performed using IBM SPSS version 24 (IBM Crop., Armonk, NY, USA).

To the best of our knowledge, no studies have been conducted to estimate the completion and acceptance rates of AD among the general population of Hong Kong. Based on our experience in conducting population based telephone surveys [29], we anticipated that a sample size of 2000 would be sufficient for estimating the prevalence of completion and acceptance of AD with reasonable precision. In fact, a sample size of 2000 would allow us to estimate the prevalence rate with a margin of error (precision) of at most ±2.2% at a 5% level of significance.

## 3. Results

### 3.1. Characteristics of the Participants

**Figure 1:**
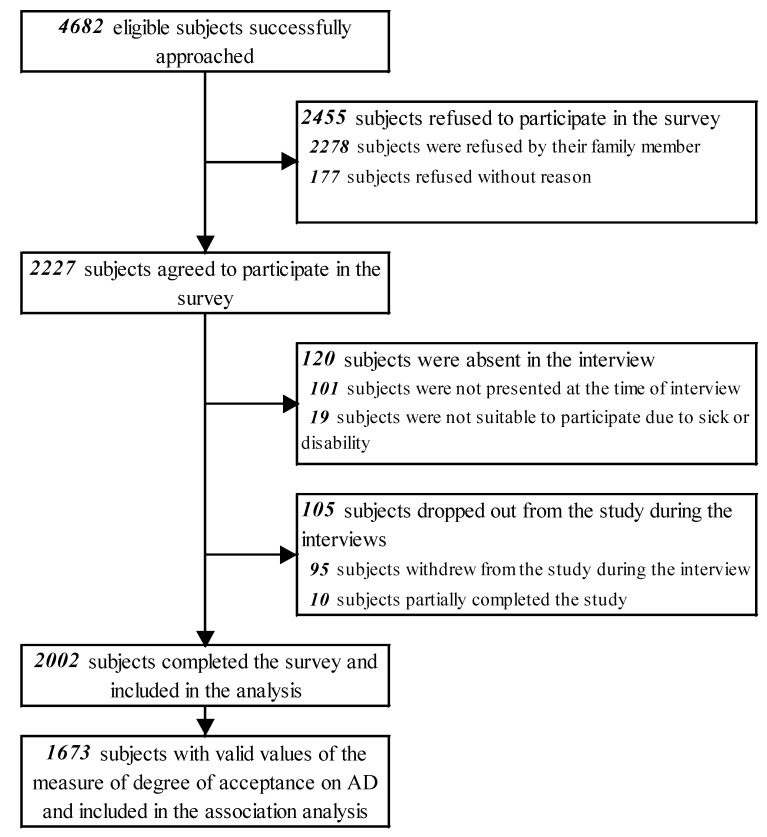
Flowchart of the study.

4682 eligible subjects were approached, among them 2227 subjects agreed to participate. During the survey, 225 subjects were either absent from the interviews or withdrew from the study. In total, 2002 participants completed the survey (response rate: 42.8%; cooperation rate: 43.7%). Figure 1 shows the flowchart of the study. The characteristics of the participants are shown in Table 1. All participants were Chinese. Our sample had more females (55.8%) and older (58.6% aged 45 or above) participants. The majority had received senior secondary education or above (70.2%). More than half were married (58.5%) and employed (53.6%). A large proportion of the participants did not have any religion (78.9%). In terms of their self-perceptions and health status, the majority had good self-rated health (62.4%). Most had not had any serious disease or cancer (94.6%) or had a family history of serious diseases or cancers (80.0%). More than half (59.4%) were optimistic. Most agreed that patients’ wishes and decisions should be respected (86.1%). Our sample had a comparable distribution of participant characteristics, including gender (54.9% females), age (55.2% aged 45 or above), and self-rated health (69.3% good) with the adult population of Hong Kong [19,30]. However, our sample was healthier than the general population of Hong Kong adults, of which 39.6% were suffering from one or more chronic conditions [30].

### 3.2. Prevalence and Perception of AD

The prevalence of AD is shown in Table 2. Less than one-fifth (18.4%) of the participants had heard about AD. Only 11 (0.5%) participants had made AD. However, the majority of those who had heard of AD had made or intended to make AD (80.2%). 

Regarding the perceptions related to AD (Table 3), the majority (82.1%) agreed that AD is a basic human right. The majority (72.7%) also thought that the promotion of AD in the community was inadequate. For the discussion of AD, the majority (77.8%) agreed that patients should have a clear mind and be mentally prepared when considering making an AD, that healthcare professionals should possess good communication skills when discussing making an AD with patients (75.7%), and that family members of the patient should be engaged in discussions for making AD (71.8%).

### 3.3. Factors Associated with Degree of Acceptance on AD Completion

The acceptance was measured by the summed score of the seven items concerning making AD in various scenarios (Table 4), using a scale with scores ranging from 0 to 100. A total of 1673 participants with a valid summed score of the level of acceptance on AD were included in the analysis to identify factors associated with the score. Using multivariable regression, we found that (1) employment status, (2) religion, (3) self-perceived level of optimism, and (4) the level of agreement that patients’ will and decisions should be respected were significantly and independently associated with the level of acceptance on AD completion (Table 5). Compared with the employed, students were significantly associated with a lower level of acceptance (regression coefficient (B): −5.89, standard error (SE): 1.41, *p* < 0.001). With reference to those without any religion, a higher degree of AD acceptance was significantly associated with those believing in Christianity (B: 5.12, SE: 1.32, *p* < 0.001), Catholicism (B: 4.78, SE: 2.37, *p* = 0.044), and Buddhism (B: 8.19, SE: 1.85, *p* < 0.001). Compared with participants perceiving themselves as pessimistic (rated 1-3 on the seven-point scale for the item assessing optimism), increasing levels of acceptance on AD completion were found among those who were becoming more optimistic (B ranged from 4.32 to 9.49, all *p* < 0.001). Moreover, a higher level of agreement by participants on the statement that patients’ wishes and decisions should be respected was found to be associated with a higher the level of acceptance on AD completion, when compared with those that disagreed with the statement (B ranging from 6.22 to 20.11, all *p* < 0.05).

## 4. Discussion

Our study revealed that the general population of Hong Kong exhibits a rather low prevalence of AD completion, with only 0.5% of our survey participants having completed AD previously. Previous studies showed that the prevalence of AD in regions worldwide ranged from 7% in the Netherlands to 34% in Maryland in the US [1,2,6,13,31]. However, in our study, 80.2% of the participants who had previously heard of AD had made or intended to make AD, implying that the majority of the participants accepted the idea of making an AD. This is supported by our finding that the majority of the participants agreed that AD is a basic human right, with 82.1% of the participants rated 5–7 for the item on the seven-point rating scale. This finding is consistent with that from a previous telephone survey in Hong Kong, with 73.9% of the participants sampled from the general population agreeing with the concept of AD after its explanation to the participants [25]. Other studies also suggested that Chinese patients with chronic diseases, such as cancer and dementia, are open to the idea of AD [32,33]. The large discrepancy in the rate of AD acceptance and that of AD completion may be explained by the inadequacy in the promotion of AD among the public. A large proportion of the participants (72.7%) reported the inadequacy of AD promotion in the community. A local telephone survey revealed that half of the participants preferred to receive comfort care without the use of any means to extend their lives, as the concept of AD was not explained to the participants in that survey [34]. A lack of awareness on AD, lack of familiarity with it, and procrastination were frequently highlighted as the reasons of low AD completion rate in previous studies [1,2,6,35]. Public education to disseminate the concept of AD is needed to raise public awareness on AD, as being asked to complete AD and receiving information or explanation about AD were found to predict AD completion [3,36]. 

The role of Chinese culture on the effectiveness of communication for disseminating the concept of AD is highlighted in this study. Our results showed that the majority of the participants agreed that individuals should have a clear mind and be mentally prepared when considering making AD (77.8%), that healthcare professionals should possess good communication skills when discussing the completion of AD with patients (75.7%), and that family members of patients should be engaged in the discussion of making AD (71.8%). These data demonstrate the importance of good communication between patients and healthcare professionals and family involvement in AD discussion for Chinese people. A review of hospital records of patients with advanced chronic obstructive pulmonary disease in Hong Kong showed that the most common decision making approach of life-sustaining treatment involved the participation of physicians, patients, and family members [37]. Asking for relatives’ advice was one of the significant independent predictors for the preference of AD among Chinese nursing home residents [38]. A systematic review pointed out a role of family in affecting the preference of advance care planning and AD among Chinese patients [39]. However, it was reported that the views of family members may hinder AD completion. In a study of Chinese patients with dementia, family caregivers were found to have poor knowledge on life-sustaining treatments, and that they may make decisions on patient care based on their own views without considering the patients’ opinions [32]. Although they may consider the possibility of saving the patients’ life and maintaining their quality of life, a sense of guilt, helplessness, and uncertainty on their decisions was reported by these caregivers after EOL decision making [40]. This may affect the future decisions they make regarding EOL care for the patients. Hence, education of family members on AD and their involvement in AD communication among the Chinese population is of utmost importance.

In our multivariable regression analysis, (1) employment status, (2) religion, (3) self-perceived level of optimism, and (4) the level of agreement that patients’ wishes and decisions should be respected were found to be significantly and independently associated with the degree of acceptance on AD completion. For employment status, student status was significantly associated with a lower degree of acceptance. This may be due to a lack of knowledge on EOL and preparation among university students. Previous studies reported that medical students generally possess poor knowledge on EOL [41,42]. A survey with Hong Kong medical students showed that only 30% of the students knew about AD and 90% felt that they had inadequate knowledge on AD and were unprepared to deal with AD and EOL issues [41]. The lack of knowledge on EOL is also common among medical students in the US [42]. In Hong Kong, the issue of life and death is only included in the curriculum of healthcare-related disciplines, such as medicine, nursing, and social work, but not in that of other disciplines, which implies that students from other disciplines may possess even poorer knowledge on EOL than medical students. Therefore, the enhancement of education on EOL should be considered, as it was found to change the attitudes of university students [43,44], especially those who possess no previous experience in dealing with patient death and those aged between 18–22 [43]. This also helps increase their preparedness in delivering EOL care [45,46]. Concerning religion, a higher degree of acceptance was significantly associated with those with beliefs in Christianity, Catholicism, and Buddhism. In contrast, people having no religious beliefs had a significantly lower degree of AD acceptance compared with Christians, Catholics, and Buddhists. Both Christians and Catholics focus on the preparation of repentance and going to heaven when they approach death. Thus, Christian and Catholic patients would consider that a prolonged life while bearing with the end-of-life sufferings appears undesirable [47]. In addition, Buddhists believe in the ubiquity and inevitability of death [48], making them more willing to accept death and discuss EOL care. A previous study involving Chinese subjects found that people without religion had lower attitudes towards death [49], which contributed to their avoidance of AD completion [50]. Being optimistic was found to increase the degree of acceptance of AD in our study. Optimistic individuals may be more mentally prepared to discuss AD, as demonstrated by a previous study showing individuals who were optimistic experienced lower levels of psychological distress when approaching death [51]. Furthermore, a high level of agreement that patients’ wishes and decisions should be respected is associated with the positive attitudes towards AD [28], and this contributes to the increased likelihood in their AD acceptance and completion [36]. AD promotion using an interactive approach with multiple reinforcement sessions can provide tailor-made information to enhance their positive attitude towards EOL and thus increase the completion rate of AD [52,53]. 

Our study identified a considerable proportion of “don’t know/unsure” in the participants’ responses to the items in the section of perceptions related to AD and the level of agreement in making AD in various scenarios in the questionnaire, with the proportion ranging from 7.4% to 21.2% and 15.0% to 20.1%, respectively. The proportion of “don‘t know/unsure” in the former section may be due to the participants’ lack of knowledge in AD, as more than 80% of the participants have never heard of AD in Hong Kong. It would be difficult for them to indicate their perceptions related to AD when they possess poor knowledge on AD. For the latter section, as the majority of participants had not had any serious disease or cancer (94.6%) or had a family history of such diseases (80.0%), they may not have the experience in dealing with their poor physical conditions, which may lead to an increase in their difficulty to consider whether they prefer to make AD under such conditions.

The strengths of our study include the use of random-digit dialing to conduct the survey with a large sample. This helps enhance the representativeness of the sample and thus the generalizability of the study findings. The interviews were conducted at night to avoid selection bias towards the non-working population. However, our study is limited by the cross-sectional nature of the study, which hampers the identification of the causal relationships between the predictors and AD completion. The use of telephone calls for conducting the interviews may have excluded eligible participants who do not possess a phone, although the number of people in Hong Kong who do not possess a phone is likely to be very low. Further, the low response rate in our survey may have introduced bias to our study, but the reasons of refusal to take part in the survey cannot be explored due to the nature of telephone surveys. Nevertheless, less than 5% of the participants refused to continue with the interview during the survey, implying that the survey topic is of a high level of acceptance to the participants. Although our study sample was comparable to the general population of Hong Kong adults in terms of gender, age, and self-rated health, our sample was healthier than the general adult population of Hong Kong. However, we cannot exclude the possibility of the existence of self-reported bias and socially desirable responses in the survey.

## 5. Conclusions

To conclude, our study revealed an extremely low prevalence of AD, but high acceptance of AD, in the Hong Kong general population. The completion of AD can be promoted by community-based AD promotion programs to raise public awareness on AD concepts. More EOL education should be made available to university students to increase their preparedness in the delivery of EOL care, and the involvement of family members is key to success in AD communication among the Chinese population. 

## Figures and Tables

**Table 1:** Characteristics of participants (*N* = 2002).

Characteristics	*N* (%)
Socio-demographic characteristics	
Sex	
Male	885 (44.2)
Female	1117 (55.8)
Age (years)	
18–24	243 (12.1)
25–34	287 (14.3)
35–44	298 (14.9)
45–54	365 (18.2)
55–64	384 (19.2)
≥65	425 (21.2)
Education	
No formal education	88 (4.4)
Primary school	229 (11.4)
Junior secondary school	259 (12.9)
Senior secondary school	687 (34.3)
Tertiary or above	719 (35.9)
Refused to answer	20 (1.0)
Marital status	
Married	1172 (58.5)
Single	692 (34.6)
Divorced/separated/widowed	114 (5.7)
Refused to answer	24 (1.2)
Employment status	
Employed	1073 (53.6)
Unemployed	97 (4.8)
Retired	415 (20.7)
Homemaker	219 (10.9)
Students	168 (8.4)
Others/refused to answer	30 (1.5)
Religion	
None	1579 (78.9)
Christianity	186 (9.3)
Catholicism	52 (2.6)
Buddhism	98 (4.9)
Chinese folk religion	41 (2.0)
Refused to answer	46 (2.3)
Monthly household income (HK$)	
<10,000	242 (12.1)
10,000–19,999	314 (15.7)
20,000–29,999	469 (23.4)
30,000–59,999	413 (20.6)
≥60,000	204 (10.2)
Don’t know/refused to answer	360 (18.0)
Self-perceptions and health status	
Self-rated health	
Good	1250 (62.4)
Average	654 (32.7)
Bad	89 (4.4)
Don’t know/unsure	9 (0.4)
Ever had a serious disease or cancer	
No	1894 (94.6)
Yes	99 (4.9)
Don’t know/unsure	9 (0.4)
Family history of serious diseases or cancers	
No	1602 (80.0)
Yes	385 (19.2)
Don’t know/unsure	15 (0.7)
Playing a key role in family	
No	594 (29.7)
Yes	1288 (64.3)
Unsure	120 (6.0)
Self-perceived level of optimism [1 to 7; 1 = very pessimistic, 7 = very optimistic]	
1–3	124 (6.2)
4	316 (15.8)
5	620 (31.0)
6	374 (18.7)
7	194 (9.7)
Don’t know/unsure	374 (18.7)
Level of agreement that patients’ wishes and decisions should be respected [1 to 7; 1 = strongly disagree, 7 = strongly agree]	
1–3	46 (2.3)
4	124 (6.2)
5	371 (18.5)
6	614 (30.7)
7	738 (36.9)
Don’t know/unsure	109 (5.4)

HK$: Hong Kong dollar. Data are presented as frequency (%).

**Table 2:** Prevalence of advance directives (*n* = 2002).

Prevalence of AD	*n* (%)
Ever heard about advance directives	
No	1629 (81.4%)
Yes	368 (18.4%)
Can’t remember	5 (0.2%)
Had made advance directives	
Had not heard of advance directives/can’t remember	1634 (81.6%)
No	357 (17.8%)
Yes	11 (0.5%)
Had made or intend to made advance directives	
Had not heard of advance directives/can’t remember	1634 (81.6%)
No	39 (1.9%)
Yes	295 (14.7%)
Don’t know/unsure	34 (1.7%)

Data are presented as frequency (%).

**Table 3:** Perceptions related to advance directives (*n* = 2002).

Perceptions Related to AD	*n* (%)							
	Rating							
	1	2	3	4	5	6	7	Don’t Know/Unsure
1. Level of agreement that (AD is a basic human right) [1 to 7; 1 = strongly disagree, 7 = strongly agree]	6 (0.3)	7 (0.3)	55 (2.7)	141 (7.0)	534 (26.7)	573 (28.6)	537 (26.8)	149 (7.4)
2. Level of agreement that (advance directives should encompass basic nursing care, including pain relief and wound cleansing etc) [1 to 7; 1 = strongly disagree, 7 = strongly agree]	73 (3.6)	77 (3.8)	125 (6.2)	288 (14.4)	493 (24.6)	314 (15.7)	343 (17.1)	289 (14.4)
3. Level of agreement that (healthcare professionals should carry out advance directives by legislation) [1 to 7; 1 = strongly disagree, 7 = strongly agree]	23 (1.1)	21 (1.0)	86 (4.3)	281 (14.0)	513 (25.6)	311 (15.5)	450 (22.5)	317 (15.8)
4. (Adequacy of promotion on advance directive in the community) [1 to 7; 1 = very inadequate, 7 = very adequate]	431 (21.5)	569 (28.4)	456 (22.8)	207 (10.3)	109 (5.4)	33 (1.6)	18 (0.9)	179 (8.9)
4. Level of agreement that (patients should have a clear mind and be mentally prepared when considering making advance directives) [1 to 7; 1 = strongly disagree, 7 = strongly agree]	5 (0.2)	8 (0.4)	13 (0.6)	74 (3.7)	213 (10.6)	469 (23.4)	876 (43.8)	344 (17.2)
5. Level of agreement that (healthcare professionals should have good communication skills when discussing making advance directives with patients) [1 to 7; 1 = strongly disagree, 7 = strongly agree]	9 (0.4)	6 (0.3)	32 (1.6)	54 (2.7)	292 (14.6)	522 (26.1)	701 (35.0)	386 (19.3)
6. Level of agreement that (in addition to healthcare professionals and patients, family members of patient should engage in discussing making advance directives) [1 to 7; 1 = strongly disagree, 7 = strongly agree]	25 (1.2)	16 (0.8)	67 (3.3)	91 (4.5)	313 (15.6)	427 (21.3)	699 (34.9)	364 (18.2)
7. Level of agreement that (records throughout the process of discussions and making decisions of advance directives with patients should be kept) [1 to 7; 1 = strongly disagree, 7 = strongly agree]	10 (0.5)	8 (0.4)	28 (1.4)	65 (3.2)	274 (13.7)	513 (25.6)	680 (34.0)	424 (21.2)

AD: advance directives. Data are presented as frequency (%).

**Table 4:** Level of agreement in making advance directives in various scenarios (*n* = 2002).

Level of Agreement [Range: 1 to 7; 1 = Strongly Disagree, 7 = Strongly Agree]	*n* (%)							
	Rating							
	1	2	3	4	5	6	7	Don’t Know/Unsure
1. (When my health condition is serious enough to have no effective treatment)	32 (1.6)	13 (0.6)	68 (3.4)	95 (4.7)	313 (15.6)	582 (29.1)	598 (29.9)	301 (15.0)
2. (In order to reduce the physical and psychological burden of family members)	12 (0.6)	16 (0.8)	47 (2.3)	95 (4.7)	251 (12.5)	649 (32.4)	631 (31.5)	301 (15.0)
3. (When considering side effects and adverse effects on quality of life in receiving survival treatments, such as mechanical ventilating and tube feeding)	28 (1.4)	42 (2.1)	67 (3.3)	196 (9.8)	395 (19.7)	443 (22.1)	492 (24.6)	339 (16.9)
4. (If healthcare professionals can provide you clear explanation and recommendation on advance directives)	15 (0.7)	11 (0.5)	41 (2.0)	100 (5.0)	363 (18.1)	536 (26.8)	582 (29.1)	354 (17.7)
5. (If there is effective communication and coordination among healthcare professionals at different institutes to execute your decisions)	169 (8.4)	192 (9.6)	39 (1.9)	83 (4.1)	347 (17.3)	383 (19.1)	444 (22.2)	345 (17.2)
6. (If you can have thorough discussion and follow-up with health professionals about advance directives)	54 (2.7)	63 (3.1)	85 (4.2)	144 (7.2)	363 (18.1)	500 (25.0)	433 (21.6)	360 (18.0)
7. (If advance directive is a legally binding instrument)	74 (3.7)	176 (8.8)	143 (7.1)	154 (7.7)	340 (17.0)	322 (16.1)	390 (19.5)	403 (20.1)

AD: advance directives. Data are presented as frequency (%).

**Table 5:** Factors associated with the degree of acceptance on completing advance directives (*n* = 1673).

Factors	Univariate Analysis		Multivariable Analysis		
	Mean (SD)	*p*-Value	B	SE	*p*-Value
Socio-demographic characteristics					
Sex					
Male (ref)	74.8 (16.7)	0.682	NR	NR	NR
Female	75.2 (17.9)				
Age (years)					
18–24 (ref)	70.0 (16.8)	<0.001	NR	NR	NR
25–34	73.6 (15.8)				
35–44	75.6 (17.8)				
45–54	77.6 (15.9)				
55–64	77.6 (16.3)				
≥65	73.8 (20.2)				
Education					
No formal education (ref)	67.8 (21.0)	0.019	NR	NR	NR
Primary school	73.3 (19.2)				
Junior secondary school	74.3 (15.8)				
Senior secondary school	76.4 (16.8)				
Tertiary or above	74.7 (17.8)				
Marital status					
Single/divorced/separated/ widowed (ref)	73.1 (17.5)	<0.001	NR	NR	NR
Married	76.4 (17.2)				
Employment status					
Employed (ref)	75.9 (16.6)	<0.001			
Unemployed	74.0 (13.6)		0.150	1.956	0.939
Retired	74.9 (19.9)		0.039	1.110	0.972
Homemaker	76.1 (17.7)		0.455	1.361	0.738
Students	68.6 (17.4)		−5.890	1.411	<0.001
Religion					
None (ref)	73.5 (17.4)	<0.001			
Christianity	80.9 (16.0)		5.124	1.316	<0.001
Catholicism	80.8 (18.8)		4.775	2.368	0.044
Buddhism	82.4 (15.2)		8.192	1.850	<0.001
Chinese folk religion	75.7 (19.1)		1.416	3.371	0.675
Monthly household income (HK$)					
<10,000 (ref)	73.0 (18.8)	0.002	NR	NR	NR
10,000–19,999	72.3 (17.2)				
20,000–29,999	74.2 (16.3)				
30,000–59,999	76.5 (17.8)				
≥60,000	78.0 (17.7)				
Don’t know/refused to answer	75.7 (16.9)				
Health status and self-perceptions					
Self-rated health					
Good (ref)	74.9 (16.6)	0.176	NR	NR	NR
Average	75.8 (18.2)				
Bad	71.2 (21.6)				
Q3. Ever had a serious disease or cancer					
No (ref)	74.9 (17.3)	0.075	NR	NR	NR
Yes	78.5 (18.7)				
Q4. Family history of serious diseases or cancers					
No (ref)	74.5 (17.2)	0.032	NR	NR	NR
Yes	76.8 (18.0)				
Q2. Play a key role in family					
No/unsure (ref)	73.4 (17.0)	0.006	NR	NR	NR
Yes	75.8 (17.6)				
Q24. Self-perceived level of optimism [1 to 7; 1 = very pessimistic, 7 = very optimistic]					
1–3 (ref)	67.9 (18.2)	<0.001			
4	72.3 (17.8)		4.319	1.782	0.015
5	75.1 (15.8)		6.359	1.648	<0.001
6	79.0 (15.2)		8.811	1.736	<0.001
7	80.3 (19.0)		9.493	1.912	<0.001
Don’t know/unsure	66.0 (21.0)		−2.127	2.321	0.360
Q5. Level of agreement that patients’ wishes and decisions should be respected [1 to 7; 1 = strongly disagree, 7 = strongly agree]					
1–3 (ref)	58.2 (22.6)	<0.001			
4	64.3 (18.6)		6.224	3.093	0.044
5	71.8 (14.6)		12.820	2.735	<0.001
6	74.5 (15.0)		15.847	2.669	<0.001
7	79.8 (17.8)		20.112	2.645	<0.001
Don’t know/unsure	66.6 (19.5)		10.061	3.617	0.005

ref: reference group of categorical variable; B: regression coefficient; SE: standard error of the regression coefficient; NR: not retained in backward multivariable regression.

## Supplementary Materials

The following are available online at http://www.mdpi.com/1660-4601/16/3/365/s1, Table S1: The survey questionnaire.
