# Supplementary material for: Prevalence, Perception, and Predictors of Advance Directives among Hong Kong Chinese: A Population-Based Survey
Source: Int J Environ Res Public Health. 2019 Jan 28;16(3):365. doi: 10.3390/ijerph16030365 (PMC6388376; doi:10.3390/ijerph16030365)
Supplement: Supplementary file 1 [file ijerph-16-00365-s001.pdf]

Prevalence, Perception, and Predictors of Advance Directives among Hong Kong Chinese: A Population-based Survey

Carmen W. H. Chan , Martin M. H. Wong , Kai Chow Choi , Helen Y. L. Chan , Amy Y. M. Chow , Raymond S. K. Lo and Michael M. K. Sham

Supplementary Material: Table S1. The survey questionnaire.

PREVALENCE, PERCEPTION AND PREDICTORS OF ADVANCE DIRECTIVES AMONG HONG KONG CHINESE: A POPULATION-BASED SURVEY

PART I: SELF-PERCEPTIONS AND HEALTH STATUS

Q1 「WHAT DO YOU THINK ABOUT YOUR PRESENT HEALTH CONDITION? IS IT VERY GOOD, GOOD, FAIR, POOR, OR VERY POOR?」

1. Very good
2. Good
3. Fair
4. Poor
5. Very poor

8. Don't know / unsure
9. Refuse to answer

Q2「HAVE YOU EVER HAD A SERIOUS DISEASE (E.G. STROKE, CANCER, HEART DISEASE OR RENAL DISEASE)? 」

1. Yes
2. No

8. Don't know / unsure
9. Refuse to answer

Q3「HAVE YOUR FAMILY MEMBERS EVER HAD A SERIOUS DISEASE (E.G. STROKE, CANCER, HEART DISEASE OR RENAL DISEASE)? 」

1. Yes
2. No

8. Don't know / unsure
9. Refuse to answer

Q4「ARE YOU PLAYING A KEY ROLE IN YOUR FAMILY (E.G. BREAD WINNER, CARER ETC)? 」

1. Yes
2. No

8. Don't know / unsure
9. Refuse to answer

Q5「DO YOU THINK YOU ARE AN OPTIMISTIC PERSON? IF ONE STANDS FOR『VERY PESSIMISTIC』 TO SEVEN FOR『VERY OPTIMISTIC』, WHAT WILL YOUR RATING BE? 」

1. 1（Very pessimistic）
2. 2
3. 3
4. 4
5. 5
6. 6

88. Don't know / unsure
99. Refuse to answer

7. 7（Very optimistic）

Q6「WHAT IS YOUR LEVEL OF AGREEMENT THAT PATIENTS' WISHES AND DECISIONS SHOULD BE RESPECTED? IF ONE STANDS FOR『STRONGLY DISAGREE』TO SEVEN FOR 『STRONLY AGREE』, WHAT WILL YOUR RATING BE? 」

1. 1（Strongly disagree）
2. 2
3. 3
4. 4
5. 5
6. 6
7. 7（Strongly agree）

88. Don't know / unsure
99. Refuse to answer

PART II: PREVALENCE OF ADVANCE DIRECTIVES

Q7「『ADVANCE DIRECTIVES』 IS A MEDICAL DOCUMENT TO ENABLE PATIENTS TO INDICATE ADVANCED REFUSAL OF MEDICAL TREATMENT AND DIRECTIONS ON THE KIND OF LIFE-SUSTAINING TREATMENTS SUCH AS ARTIFICAL VENTILATION AND CARDIOPULMONARY RESUSCITATION TO BE WITHHELD OR WITHDRAWN WHEN HE OR SHE IS NO LONGER MENTALLY CAPABLE OF MAKING HEALTHCARE DECISIONS. BEFORE THIS SURVEY, HAVE YOU EVER HEARD ABOUT『ADVANCE DIRECTIVES』? 」

1. Yes【Go to Q8】
2. No【Skip Part II】

8. Can't remember【Skip Part II】
9. Refuse to answer【Skip Part II】

【This question only asks participants who have heard about『Advance Directives』(Q7=1)】
Q8「HAVE YOU MADE『ADVANCE DIRECTIVES』? 」

1. Yes【Skip Part II】
2. No【Go to Q9】

9. Refuse to answer【Skip Part II】

【This question only asks participants who have not made『Advance Directives』(Q8=2)】
Q9 「HAVE YOU PLANNED TO MAKE『ADVANCE DIRECTIVES』IN THE FUTURE? 」

1. Yes
2. No

8. Don't know / unsure
9. Refuse to answer

PART III: PERCEPTIONS RELATED TO ADVANCE DIRECTIVES

「THE FOLLOWING ARE SOME PERCEPTIONS RELATED TO 『ADVANCE DIRECTIVES』, DO YOU AGREE WITH THEM? IF ONE STANDS FOR 『VERY DISAGREE』 TO SEVEN FOR 『VERY AGREE』, FROM ONE TO SEVEN, WHAT WILL YOUR RATING BE? 」

Q10 「『ADVANCE DIRECTIVES』 IS A BASIC HUMAN RIGHT FOR EVERYONE, DO YOU AGREE? FROM ONE TO SEVEN, WHAT WILL YOUR RATING BE?」

1. 1（Strongly disagree） 88. Don't know / unsure
2. 2 99. Refuse to answer
3. 3
4. 4
5. 5
6. 6
7. 7（Strongly agree）

Q11「IF 『ADVANCE DIRECTIVES』 ENCOMPASS BASIC NURSING CARE SUCH AS PAIN RELIEF AND WOUND CLEANSING, WOULD YOU AGREE? FROM ONE TO SEVEN, WHAT WILL YOUR RATING BE?」

1. 1（Strongly disagree） 88. Don't know / unsure
2. 2 99. Refuse to answer
3. 3
4. 4
5. 5
6. 6
7. 7（Strongly agree）

Q12「IF ONE STANDS FOR 『STRONGLY DISAGREE』 TO SEVEN FOR 『STRONGLY AGREE』, TO WHAT EXTENT WOULD YOU AGREE THAT HEALTHCARE PROFESSIONALS SHOULD CARRY OUT 『ADVANCE DIRECTIVES』 BY LEGISLATION? FROM ONE TO SEVEN, WHAT WILL YOUR RATING BE?」

1. 1（Strongly disagree） 88. Don't know / unsure
2. 2 99. Refuse to answer
3. 3
4. 4
5. 5
6. 6
7. 7（Strongly agree）

Q13「DO YOU THINK THERE IS ADEQUATE PROMOTION OF 『ADVANCE DIRECTIVES』 IN THE COMMUNITY? IF ONE STANDS FOR 『VERY INADEQUATE』 TO SEVEN FOR 『VERY ADEQUATE』, FROM ONE TO SEVEN, WHAT WILL YOUR RATING BE?」

1. 1（Very inadequate） 88. Don't know / unsure
2. 2 99. Refuse to answer
3. 3
4. 4
5. 5
6. 6
7. 7（Very adequate）

Q14「WHEN CONSIDERING MAKING 『ADVANCE DIRECTIVES』, PATIENTS SHOULD HAVE A CLEAR MIND AND BE MENTALLY PREPARED, DO YOU AGREE? FROM ONE TO SEVEN, WHAT WILL YOUR RATING BE?」

1. 1（Strongly disagree） 88. Don't know / unsure
2. 2 99. Refuse to answer
3. 3
4. 4
5. 5
6. 6
7. 7（Strongly agree）

Q15「WHEN DISCUSSING MAKING 『ADVANCE DIRECTIVES』 WITH PATIENTS, HEALTHCARE PROFESSIONALS SHOULD POSSESS GOOD COMMUNICATION SKILLS, DO YOU AGREE? FROM ONE TO SEVEN, WHAT WILL YOUR RATING BE?」

1. 1（Strongly disagree） 88. Don't know / unsure
2. 2 99. Refuse to answer
3. 3
4. 4
5. 5
6. 6
7. 7（Strongly agree）

Q16「WHEN DISCUSSING MAKING 『ADVANCE DIRECTIVES』, FAMILY MEMBERS OF PATIENTS SHOULD BE ENGAGED IN THE DISCUSSION IN ADDITION TO HEALTHCARE PROFESSIONALS AND PATIENTS. DO YOU AGREE? FROM ONE TO SEVEN, WHAT WILL YOUR RATING BE?」

1. 1（Strongly disagree） 88. Don't know / unsure

2. 2 99. Refuse to answer
3. 3
4. 4
5. 5
6. 6
7. 7（Strongly agree）

Q17「WHEN DISCUSSING MAKING 『ADVANCE DIRECTIVES』, RECORDS THROUGHOUT THE PROCESSES OF DISCUSSION AND DECISION MAKING ON AD COMPLETION WITH PATIENTS SHOULD BE KEPT, DO YOU AGREE? FROM ONE TO SEVEN, WHAT WILL YOUR RATING BE?」

1. 1（Strongly disagree） 88. Don't know / unsure
2. 2 99. Refuse to answer
3. 3
4. 4
5. 5
6. 6
7. 7（Strongly agree）

PART IV: MAKING ADVANCE DIRECTIVES IN VARIOUS SCENARIOS

「IF ONE STANDS FOR 『STRONGLY DISAGREE』 TO SEVEN FOR 『STRONGLY AGREE』, TO WHAT EXTENT DO YOU AGREE TO CONSIDER MAKING 『ADVANCE DIRECTIVES』 UNDER THE FOLLOWING CONDITIONS?

Q18「WHEN MY HEALTH CONDITION IS TOO SERIOUS TO BE TREATED EFFECTIVELY, I WOULD CONSIDER TO MAKE 『ADVANCE DIRECTIVES』, DO YOU AGREE? FROM ONE TO SEVEN, WHAT WILL YOUR RATING BE?」

1. 1（Strongly disagree） 88. Don't know / unsure
2. 2 99. Refuse to answer
3. 3
4. 4
5. 5
6. 6
7. 7（Strongly agree）

Q19「IN ORDER TO REDUCE THE PHYSICAL AND PSYCHOLOGICAL BURDEN OF FAMILY MEMBERS, I WOULD CONSIDER TO MAKE

『ADVANCE DIRECTIVES』, DO YOU AGREE? FROM ONE TO SEVEN, WHAT WILL YOUR RATING BE?」

1. 1（Strongly disagree） 88. Don't know / unsure
2. 2 99. Refuse to answer
3. 3
4. 4
5. 5
6. 6
7. 7（Strongly agree）

Q20「WHEN CONSIDERING SIDE EFFECTS OF LIFE-SUSTAINING TREATMENTS (SUCH AS MECHANICAL VENTILATING AND TUBE FEEDING) AND THEIR ADVERSE EFFECTS ON QUALITY OF LIFE , I WOULD CONSIDER TO MAKE 『ADVANCE DIRECTIVES』, DO YOU AGREE? FROM ONE TO SEVEN, WHAT WILL YOUR RATING BE?」

1. 1（Strongly disagree） 88. Don't know / unsure
2. 2 99. Refuse to answer
3. 3
4. 4
5. 5
6. 6
7. 7（Strongly agree）

Q21「IF HEALTHCARE PROFESSIONALS CAN PROVIDE YOU WITH CLEAR EXPLANATION AND RECOMMENDATION ON 『ADVANCE DIRECTIVES』, I WOULD CONSIDER TO MAKE 『ADVANCE DIRECTIVES』, DO YOU AGREE? FROM ONE TO SEVEN, WHAT WILL YOUR RATING BE?」

1. 1（Strongly disagree） 88. Don't know / unsure
2. 2 99. Refuse to answer
3. 3
4. 4
5. 5
6. 6
7. 7（Strongly agree）

Q22「IF THERE IS EFFECTIVE COMMUNICATION AND COORDINATION AMONG HEALTHCARE PROFESSIONALS AT DIFFERENT INSTITUTIONS (E.G. OLD AGED HOME, HOSPITAL AND AMBULENCE SERVICE) TO EXECUTE YOUR DECISIONS, I WOULD CONSIDER TO MAKE 『ADVANCE DIRECTIVES』, DO YOU AGREE? FROM ONE TO

SEVEN, WHAT WILL YOUR RATING BE?」

1. 1（Strongly disagree）
2. 2
3. 3
4. 4
5. 5
6. 6
7. 7（Strongly agree）

88. Don't know / unsure
99. Refuse to answer

Q23「IF YOU CAN HAVE THOROUGH DISCUSSIONS AND FOLLOW-UP WITH HELATHCAR PROFESSIONALS ABOUT 『ADVANCE DIRECTIVES』, I WOULD CONSIDER TO MAKE 『ADVANCE DIRECTIVES』, DO YOU AGREE? FROM ONE TO SEVEN, WHAT WILL YOUR RATING BE?」

1. 1（Strongly disagree）
2. 2
3. 3
4. 4
5. 5
6. 6
7. 7（Strongly agree）

88. Don't know / unsure
99. Refuse to answer

Q24「IF 『ADVANCE DIRECTIVES』 IS A LEGALLY BOUND DOCUMENT (E.G. UNDER LAW REGULATION REGARDING TO ITS EXECUTION), I WOULD CONSIDER TO MAKE 『ADVANCE DIRECTIVES』, DO YOU AGREE? FROM ONE TO SEVEN, WHAT WILL YOUR RATING BE?」

1. 1（Strongly disagree）
2. 2
3. 3
4. 4
5. 5
6. 6
7. 7（Strongly agree）

88. Don't know / unsure
99. Refuse to answer

PART V: SOCIO-DEMOGRAPHIC CHARACTERISTICS

「IN ORDER TO ANALYZE THE VIEWS OF PARTICIPANTS WITH VARIOUS BACKGROUNDS, I WOULD LIKE TO OBTAIN SOME DEMOGRAPHIC INFROMATMION FROM YOU.」

SEX： 1. Male 2. Female

AGE「WHICH OF THE FOLLWING AGE GROUPS DO YOU BELONG TO?」

1. 18 to 24 years old
2. 25 to 34 years old
3. 35 to 44 years old
4. 45 to 54 years old
5. 55 to 64 years old
6. 65 years old or above

9. Refuse to answer

EDU「WHAT IS YOUR EDUCATION LEVEL?」

1. No formal education（Kindergarten）
2. Primary school（Primary one to six）
3. Junior secondary school（Secondary one to three）
4. Senior secondary school（Secondary four to seven / vocational institute）
5. Tertiary or above（including associate degree / tertiary institute / polytechnic / university etc）

9. Refuse to answer

MARITAL「WHAT IS YOUR MARITAL STATUS?」

1. Married
2. Single
3. Others: separated or divorced / widowed
4. Others（Please specify）

9. Refuse to answer

WORK「WHAT IS YOUR EMPLOYMENT STATUS?」

【WORKING: INCLUDES FULL-TIME AND PART-TIME】

【NON-WORKING: ASK THE CATEGORIES -->「WHAT CATEGORY ARE YOU BELONG TO? UNEMPLOYED, RETIRED, HOMEMAKER, OR STUDENT?」】

1. Working
2. Non-working: Unemployed
3. Non-working: Retired
4. Non-working: Homemaker
5. Non-working: Student
6. Non-working: Others（Sick / Break）

9. Refuse to answer

RELIGION「DO YOU HAVE ANY RELIGIOUS BELIEFS? IF YES, WHAT IS

YOUR BELIEF?」

1. None
2. Christianity
3. Catholicism
4. Buddhism
5. Taoism
6. Chinese folk religion (e.g. Worship God, Tin Hau)
7. Others (Please specify)
9. Refuse to answer

INCOME「WHAT IS YOUR MONTHLY HOUSEHOLD INCOME?」

1. <10,000
2. 10,000 – 19,999
3. 20,000 – 29,999
4. 30,000 – 59,999
5. ≥60,000
8. Don’t know
9. Refuse to answer

「THE SURVEY IS COMPLETED. THANK YOU FOR YOUR PARTICIPATION IN THE SURVEY. GOOD BYE!」
